# Stem-loop structures in prokaryotic genomes

**DOI:** 10.1186/1471-2164-7-170

**Published:** 2006-07-04

**Authors:** Mauro Petrillo, Giustina Silvestro, Pier Paolo Di Nocera, Angelo Boccia, Giovanni Paolella

**Affiliations:** 1 CEINGE Biotecnologie Avanzate scarl Via Comunale Margherita 482, 80145 Napoli, Italy; 2 Dipartimento di Biologia e Patologia Cellulare e Molecolare, Università Federico II Via S. Pansini 5, 80131 Napoli, Italy; 3 Dipartimento SAVA Università del Molise Via De Sanctis, 86100 Campobasso, Italy; 4 Dipartimento di Biochimica e Biotecnologie Mediche, Università Federico II Via S. Pansini 5, 80131 Napoli, Italy

## Abstract

**Background:**

Prediction of secondary structures in the expressed sequences of bacterial genomes allows to investigate spontaneous folding of the corresponding RNA. This is particularly relevant in untranslated mRNA regions, where base pairing is less affected by interactions with the translation machinery. Relatively large stem-loops significantly contribute to the formation of more complex secondary structures, often important for the activity of sequence elements controlling gene expression.

**Results:**

Systematic analysis of the distribution of stem-loop structures (SLSs) in 40 wholly-sequenced bacterial genomes is presented. SLSs were searched as stems measuring at least 12 bp, bordering loops 5 to 100 nt in length. G-U pairing in the stems was allowed. SLSs found in natural genomes are constantly more numerous and stable than those expected to randomly form in sequences of comparable size and composition. The large majority of SLSs fall within protein-coding regions but enrichment of specific, non random, SLS sub-populations of higher stability was observed within the intergenic regions of the chromosomes of several species. In low-GC firmicutes, most higher stability intergenic SLSs resemble canonical rho-independent transcriptional terminators, but very frequently feature at the 5'-end an additional A-rich stretch complementary to the 3' uridines. In all species, a clearly biased SLS distribution was observed within the intergenic space, with most concentrating at the 3'-end side of flanking CDSs. Some intergenic SLS regions are members of novel repeated sequence families.

**Conclusion:**

In depth analysis of SLS features and distribution in 40 different bacterial genomes showed the presence of non random populations of such structures in all species. Many of these structures are plausibly transcribed, and might be involved in the control of transcription termination, or might serve as RNA elements which can enhance either the stability or the turnover of cotranscribed mRNAs. Three previously undescribed families of repeated sequences were found in *Yersiniae*, *Bordetellae *and *Enterococci*.

## Background

The tremendous flow of information generated by large scale genome-sequencing provided, as far as the prokaryotic world is concerned, the complete DNA sequence of over 200 bacterial strains, and more are becoming available every month. Most annotation work has been directed to the assessment of the protein repertoire encoded by a given microbe, aiming to the genome-scale reconstruction of bacterial metabolism [1], the identification of gene sets unique to pathogenic microorganisms [2,3] or the development of new vaccines [4]. The availability of massive amount of sequence data also stimulated in depth evaluation of the organization of the bacterial chromosome [5-9]. The basic organization of the genetic material (DNA curvature and stacking energy, base and oligo skews, etc.; see ref. [10]), and the presence of simple or more complex sequence repeats [11,12] have also been analyzed for most sequenced bacterial genomes.

Information associated to the folding of specific, single stranded sequence regions into secondary structures is relatively ill-defined in prokaryotes. Prediction of RNA secondary structures may show different and even contrasting results, depending on the methodologies and the genomic regions evaluated [13-15].

In bacteria, protein coding sequences may be regarded as able to be transcribed and to form predictable secondary structures, although in many instances the spontaneous folding of the corresponding mRNA may be affected by interactions with the translation machinery. Stem-loop-structures (SLSs) in RNA may in turn control transcription, as in the attenuation mechanism [16], or influence translation, as SECIS elements do for the insertion of selenocysteine at stop codons [17]. Secondary structure prediction is very effective for relatively small RNA with defined ends, especially when corroborated by phylogenetic data, but it is more ambiguous in larger RNAs, where SLSs, especially those containing short stems, are easily formed, or lost, when a sliding window is used to tentatively delimit the boundaries of a folding domain.

Longer stems significantly contribute to the formation of complex secondary structures where they affect RNA stability and functionality. Many non coding RNA structures are known to fold around a stem which delimits either a small, simple, single-strand loop or a larger, highly structured sequence. Examples are found in self-splicing introns [18], riboswitches [19], transcribed intergenic repeats such as *E.coli *BIME,* Yersinia *ERIC and *Neisseria *NEMIS sequences. In these cases the stem is often essential to the attainment of the correct secondary structure and may be directly recognized by ribonucleases [20-23]. Some predicted SLSs might also form in DNA and affect its conformation: base pairing of single stranded DNA is known to play a role in recombination, replication and transcription [24-26].

Here we present a systematic analysis of SLS distribution in prokaryotic genomes. Sequences able to fold into stem-loop structures featuring relatively large (12 or more bp) stems have been searched and analyzed in 40 wholly-sequenced bacterial chromosomes. SLSs found in searched bacterial genomes are more numerous and more stable than those randomly expected to form in sequences of comparable size and composition. The enrichment of specific SLS sub-populations may be observed within selected intergenic regions (IGRs).

## Results

### Identification of stem-loop structures (SLSs)

A relatively large number of completely sequenced bacterial genomes is currently available, from different species of medical, industrial or purely scientific interest. While for some species only one or two strains have been sequenced, for others, such as *E. coli *and *Salmonellae*, multiple variant strains have been sequenced, leading to over-representation of these sequences in available databases. For the present study, we selected a set of 40 genomes from different bacterial species (Table 1), constituting a representative sample of the prokaryotic world in terms of evolutionary distance, genome complexity and GC content.

**Table 1:** Bacterial species analyzed in this study are numbered 1 to 40. The strains used for *in silico *analyses, the size of their genomes in base pairs and their relative GC content are shown. Representative species chosen for comparative analyses are labeled a through to v.

**Division**	**Species**	**Strain**			**Genome size**	**GC%**
						
low-GC Firmicutes	*Bacillus anthracis*	Ames	1	a	5227293	35.4
	*Bacillus halodurans*	C-125	2		4202353	43.6
	*Bacillus subtilis*	168	3		4214810	43.5
	*Clostridium perfringens*	13	4		3031430	28.5
	*Clostridium tetani*	E88	5	b	2799250	28.7
	*Enterococcus faecalis*	V583	6	c	3218031	37.0
	*Lactobacillus johnsonii*	NCC533	7		1992676	34.6
	*Listeria innocua*	CLIP11262	8		3011208	37.3
	*Listeria monocytogenes*	EGD-e	9	d	2944528	37.9
	*Staphylococcus aureus*	MW2	10		2820462	32.7
	*Streptococcus pneumoniae*	TIGR4	11	e	2160837	39.6
	*Streptococcus pyogenes*	SF370	12		1852442	38.4
						
Mollicutes	*Mycoplasma genitalium*	G-37	13	f	580074	31.6
	*Mycoplasma pneumoniae*	M129	14	g	816394	39.9
	*Ureaplasma urealyticum*	serovar 3	15		751719	25.4
						
high-GC Firmicutes	*Corynebacterium diphtheriae*	NCTC13129	16		2488635	53.5
	*Mycobacterium leprae*	TN	17		3268203	57.7
	*Mycobacterium tuberculosis*	H37Rv	18	h	4411529	65.5
						
Chlamydiae	*Chlamydia pneumoniae*	AR39	19	i	1229853	40.5
	*Chlamydia trachomatis*	serovar D	20		1042519	41.2
						
Spirochaetae	*Treponema pallidum*	Nichols	21	j	1138012	52.7
						
α-Proteobacteria	*Brucella melitensis*	16 M	22	k	3294931	57.1
	*Rickettsia conorii*	Malish 7	23		1268755	32.4
	*Rickettsia prowazekii*	Madrid E	24	l	1111523	28.9
						
β-Proteobacteria	*Bordetella bronchiseptica*	RB50	25	m	5339179	68.1
	*Bordetella parapertussis*	12822	26		4773551	68.1
	*Bordetella pertussis*	Tohama I	27		4086189	67.7
	*Neisseria meningitidis*	MC58	28	n	2272351	51.4
						
γ-Proteobacteria	Buchnera	APS	29		640681	26.2
	*Escherichia coli *K12	MG1655	30	o	4639221	49.8
	*Haemophilus influenzae*	KW20 Rd	31	p	1830138	38.0
	*Pasteurella multocida*	PM70	32		2257487	40.3
	*Pseudomonas aeruginosa*	PA01	33	q	6264403	66.4
	*Pseudomonas putida*	KT2440	34		6181863	61.5
	*Salmonella typhi*	CT-18	35	r	4809037	52.0
	*Salmonella typhimurium *LT2	SGSC141	36	s	4857432	52.2
	*Vibrio cholerae*	N16961	37		4033464	47.2
	*Yersinia pestis*	CO92	38	t	4653728	47.5
						
ε-Proteobacteria	*Campylobacter jejuni*	NCTC11168	39	u	1641481	30.5
	*Helicobacter pylori*	26695	40	v	1667867	38.8

**Figure 1:**
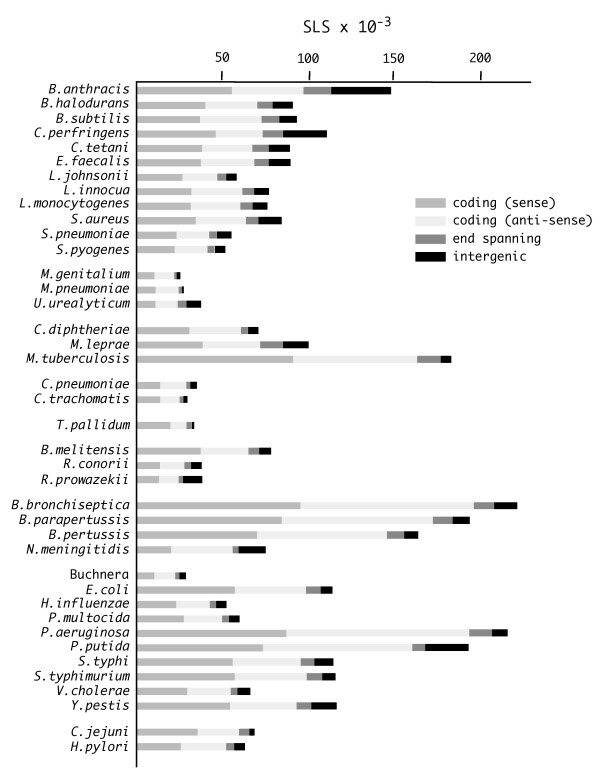
**SLSs in bacterial genomes**. The number of SLSs found in the 40 bacterial genomes listed in Table 1 is reported. SLS located completely within intergenic or coding regions are labeled as such ("sense" and "anti-sense" indicates the coding and non-coding strand, respectively); those spanning the border of coding/non-coding regions are marked as "end spanning".

Genomes of bacteria listed in Table 1 were analyzed to identify all the single-strand sequence regions able to fold into SLSs featuring double-stranded stem regions measuring at least 12 bp, and loops 5 to 100 nt long. GU base-pairs within the stem were allowed. Similarly allowed was a single mismatch or bulge located at least two matches away from the ends of the stem. These settings were chosen in order to identify both short 'canonical' stem-loop structures (i.e. with simple loops) and larger ones containing 'highly structured loops'. The number of SLSs found in each bacterial genome, grouped according to the SLS position, relatively to the boundaries of known and predicted genes annotated in the TIGR database, is reported in Fig. 1. SLSs are classified according to the following categories: a) *coding*, entirely contained within a coding sequence, located either on the sense or the anti-sense strand; b) *intergenic*, entirely located between coding sequences; c) *end-spanning*, spanning one of the ends of a coding sequence. The number of SLSs ranges from the slightly more than 20.000, in *Mycoplasmae *and other small genomes, to about 200.000, in large genomes as those featured by *Bordetellae *and *Pseudomonaceae*.

The large majority of SLSs falls within, or spans the ends of, genic regions; only about 10% of SLSs were found in IGRs. This distribution is not surprising as it reflects the high fraction (87–90%) of sequences annotated as coding in most tested genomes. In some species, however, the number of SLSs found in the IGRs was noticeably higher. In *B. anthracis*, *C. perfringens *and *N. meningitidis*, the fraction of SLSs found in non-coding sequences exceeds 20%. A slightly lower number of intergenic SLSs was found in the *P. putida *genome.

### SLSs in naturally occurring and reshuffled genomes

**Figure 2:**
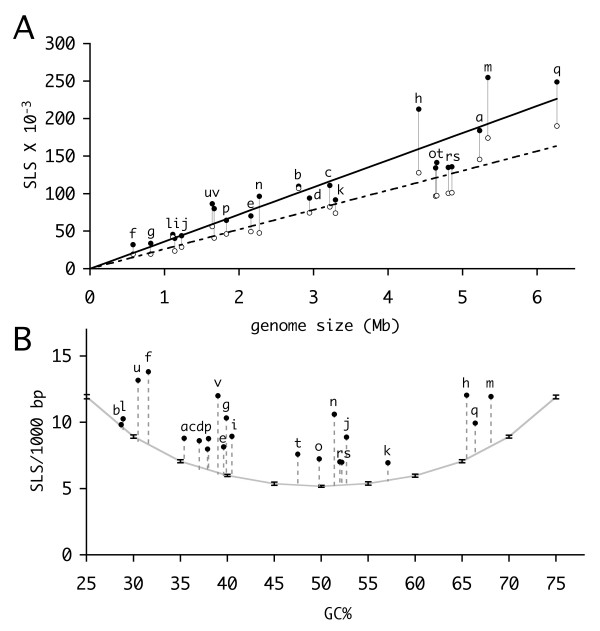
**SLSs in natural genomes and comparable random sequences**. SLSs found in natural bacterial genomes (filled circles) and random sequences of the same base composition (empty circles) are plotted against genome size in panel **A**. Filled and empty circles are connected for clarity. The same SLSs, normalized according to genome size, are plotted as a function of the GC content in panel **B**. The grey curve was obtained by determining the number of SLSs in a 1 Mbase random sequence of the indicated GC%. Each point is the average of 10 sequences, bars indicate standard deviations. Pre-filtering of SLSs was not performed. In both panels letters indicate bacterial species as listed in Table 1.

Fig. 2A reports the number of SLSs found, as a function of genome size, in the subset of 22 genomes labeled **a **to **v **in Table 1. As also shown in Fig. 1, larger genomes contain more SLSs than smaller ones, and a rough linear correspondence between genome size and SLS abundance may be observed (Fig. 2A). The same search, done on random sequences of the same length and GC content as the original genomes, produces a lower number of SLSs, linearly correlated with genome size, with the only exception of *Clostridium tetani *and *Rickettsia *genomes (Fig. 2A). As expected, for a given random genome, the number of SLSs perfectly correlates with the sequence length, when smaller fragments are tested (data not shown).

The attitude of a sequence to randomly give rise to stem-loop structures is expected to depend on a number of features, such as base composition and word frequencies. Moving away from the equally-split 25% frequency of each base, or 50% GC content, sequence complexity is reduced, and this facilitates the formation of complementary structures. This is easily seen in Fig. 2B, where SLSs found in naturally occurring genomes are plotted against GC content, after sequence length normalization. The dotted line represents SLSs found in random sequences of different GC content, all 1 Mbase long, produced by ten runs of the reshuffle tool. Variations are always within a very small (about 1%) range. As expected, random sequences stochastically give rise to a number of SLSs, which regularly grows from a minimum, for a 50% GC content, to larger numbers as GC content either decreases or increases (Fig. 2B).

**Figure 3:**
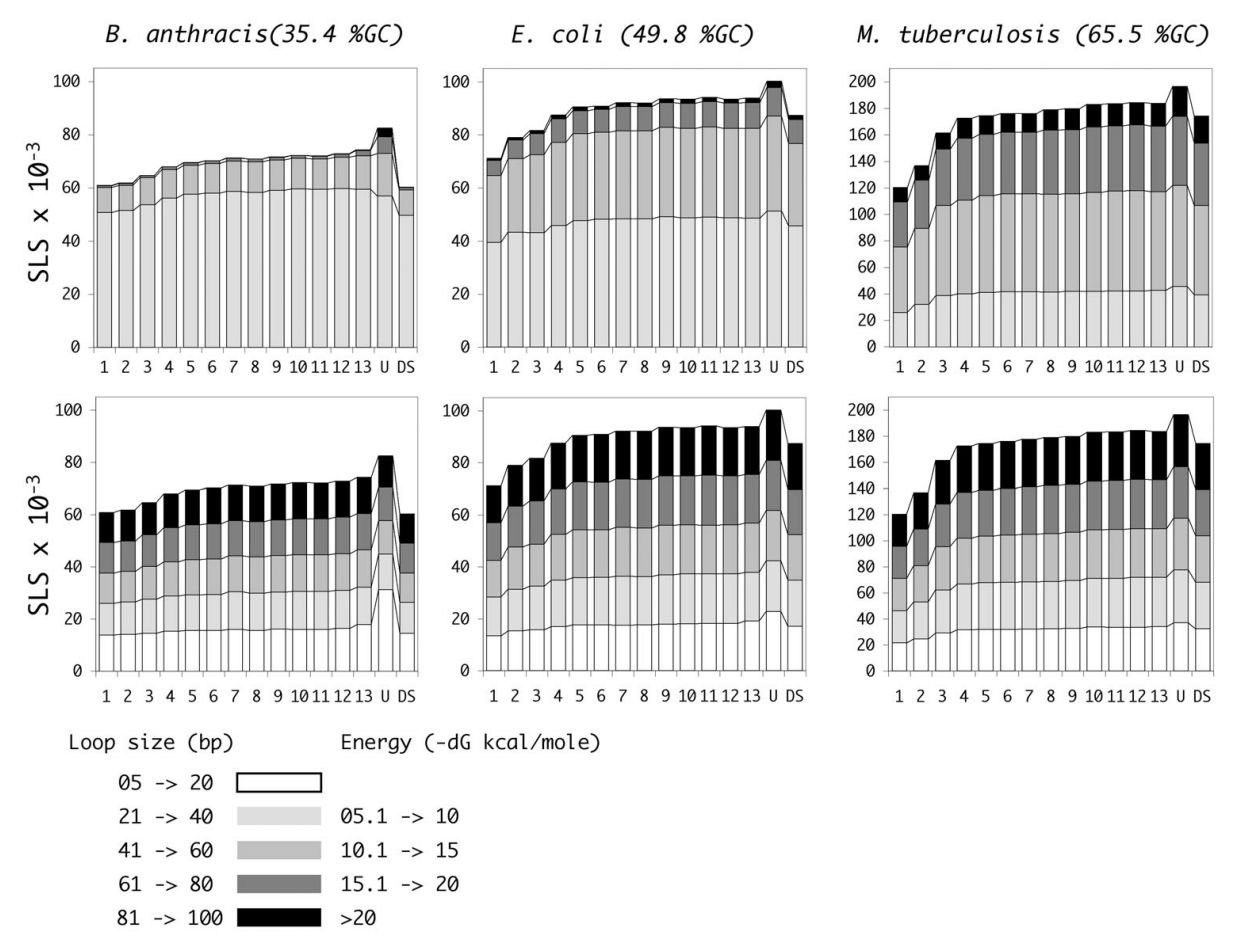
**SLSs in *B. anthracis*, *E. coli*, and *M. tuberculosis***. In the six panels, bars represent SLSs found in *B. anthracis*, *E. coli*, and *M. tuberculosis *genomes and in randomized genomes produced from them. Bars 1 to 13 refer to randomly shuffled genomes preserving the frequency of 1 to 13 nucleotide words, respectively. Bar U (Unshuffled) refers to the natural genome, while bar DS (Double Shuffle) refers to the genome shuffled preserving information about coding regions (see Results and Methods). In each column, stacked bars are used to separate SLSs of different *dG *(top panels) or loop size (bottom panels), as indicated. Only SLSs with *dG *< -5 KCal/mole were selected. Pre-filtering of SLSs was not performed. Standard deviations are always lower than 1% and are not reported in the figure.

Naturally occurring genomes feature a larger number of SLSs. With the only exception of *C. tetani*, the number of predicted SLSs is always higher, by several standard deviations, than in random sequences of comparable GC content, which is statistically significant for P < 0.0001 or lower. This indicates that some non-random component of the natural genome sequence is responsible for the larger number of SLSs, which cannot be reproduced in the shuffled sequences. Reduced complexity of the sequence may be due to consistently repeating patterns in the natural sequence, such as the tendency to prefer the use of specific di- or tri-nucleotides, or higher order words, which are not conserved in the shuffled genomes, or constraints imposed by the presence of coding regions. To test these effects, SLS found in natural genomes were compared to those found in randomized genomes, produced by shuffling while keeping constant the frequency of words of size ranging between 2 and 13 nucleotides. Shuffling genomes by only preserving word frequencies does not take into account the constraints imposed by the presence of coding regions. For this reason an alternative method (DS, double shuffle) for shuffling the natural genomes was devised, where for non-coding regions, dinucleotide frequencies are preserved, whereas for coding regions dinucleotide frequencies, encoded protein, and codon usage are all preserved, as described in reference [15]. Each randomization was repeated ten times, with very small changes in the number of SLSs found (typically <1%). The results, for three genomes of different GC content, are reported in Fig. 3, where SLSs are classified according to their stability (dG) and loop size. Progressively larger SLSs numbers are obtained by keeping the frequency of 2- to 4-nt words constant. For larger word sizes the trend appears to slow down, and subsequent increases only give rise to marginally higher numbers. Natural genomes also contain more SLSs than sequences produced according to the second, more complex, model, which distinguishes between coding and non-coding genomic regions. The differences, also in this case, are typically above four standard deviations, significant for P < 0.0001 or lower. SLS numbers obtained with this method are similar to those from genomes randomized by preserving 4- or 5-nt words. Interestingly, preserving larger k-lets ranging from 6 to 13, produces even higher numbers than the random genomes obtained by preserving codon usage.

Specific SLS subsets are selectively enriched in the natural genomes. The largest differences are observed with higher stability structures where the random component is expected to be lower. SLSs including the smallest loops (shorter than 20 bases) also appear to be more frequent in natural genomes, possibly including specific classes of RNA structures (Fig. 3).

### Identification of specific SLS groups

**Figure 4:**
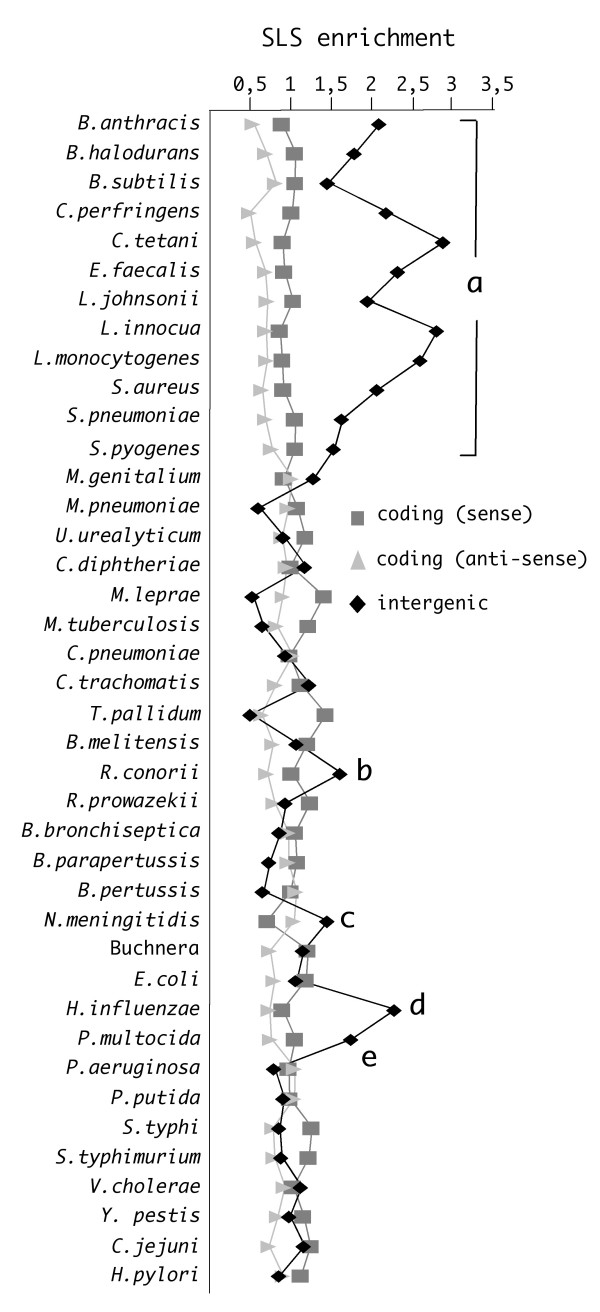
**Species-specific enrichment in intergenic SLSs**. The regional enrichment of higher stability SLSs (*dG *< -10 KCal/mole) completely located within coding (sense and anti-sense) and intergenic regions has been monitored in the 40 bacterial genomes listed in Table 1. Enrichment is expressed as the ratio of the SLS frequencies within each region to average SLS frequencies in the total genome. Letters **a **to **e **signal enrichments in intergenic SLSs observed in specific genomes (see Results).

From the previous data, it emerges that in most species the pool of predicted SLSs shows a bias towards energy levels and genome localization, which is highly indicative of the inclusion of non-random SLS sub-populations. As folding of SLSs containing larger 'loops' might produce alternative structures, possibly excluding the expected stem, minimum energy structures were predicted both freely and by imposing a constraint for SLS formation (see Methods). Most higher stability (*dG *< -10 KCal/mol) SLS-containing regions, when minimum energy structures are predicted by imposing no constraint for SLS formation, produce results within 5 KCal/mol of the SLS based structure (60 to 80% in practically all species, not shown). This indicates that, for these higher stability regions, the SLS containing structure is expected to be either the optimal or a close suboptimal structure. The relative frequency of these regions, within coding and non-coding genomic areas, was determined in the 40 bacterial genomes listed in Table 1. The results, reported in Fig. 4, were normalized to genome length and total SLS genomic frequency. Only SLSs entirely located within coding and intergenic regions were counted. An evident enrichment in intergenic SLSs can be observed in the genomes of all the low-GC firmicutes (**a **bracket in Fig. 4) and in a few proto-bacteria (**b-e**). In both *H. influenzae *and *P. multocida *(**d **and **e, **respectively, in Fig. 4), the SLS enrichment reflects the genomic over-representation of the decameric sequence, known as DUS (for DNA Uptake Sequence), which plays a role in transformation [27]. Most of the >~1000 DUS repeats found in either species are localized in intergenic spaces, and several are located next to each other in inverted orientation [27,28]. We assessed that this fraction of DUS repeats accounts for the formation of higher stability SLSs (not shown). The abundance of intergenic SLSs in the *R. conorii *and *N. meningitidis *genomes (**b **and **c**, respectively, in Fig. 4), correlates to the presence of species-specific palindromic repeats [29,30]. In contrast, the enrichment in intergenic SLSs in low-GC firmicutes cannot be explained by the presence of large repeated DNA families. In these genomes higher stability SLSs range in size from 30 to 50 nt, and show heterogeneity in both stem and loop lengths (not shown).

### AT-rich terminator-like sequences in low-GC firmicutes

**Figure 5:**
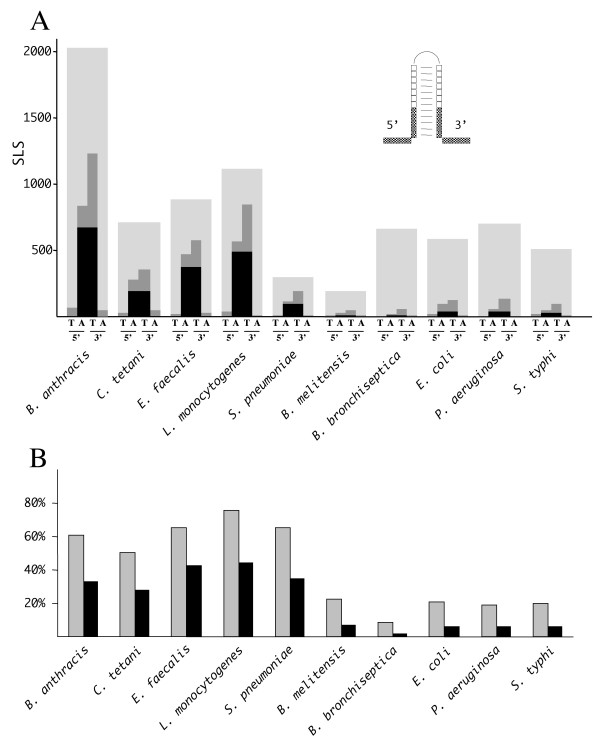
**Distribution of A- and T-runs at SLS *termini ***. Intergenic SLSs from the indicated genomes with a *dG *< -10 KCal/mole and a loop length < 30 nt, located 20 bp or less from the stop codons of CDSs (large grey bars) were screened for the presence of either A- or T-tetramers or both tetramer types at the 5' and 3' borders. Border is defined here as the 10 nt long region including, in each SLS, the first 5 nt of the stem and the 5 nt located immediately outside of the stem, either at the 5' or the 3' side (see inset in panel A). **A) **For each analyzed species, the four bars respectively indicate the number of SLSs containing 5'T-, 5'A-,3'T-and 3A'-runs of at least four identical residues. The black portions of the bars indicate the contemporary presence of both 5'As and 3'Ts. The height of the light grey regions in the background represents the total number of SLSs in the analyzed pools. **B**) The fraction of SLSs carrying 3'T-runs (grey bars) or both 5'A- and 3'T-runs (black bars) in the analyzed genomes is shown.

The analysis of higher stability, intergenic SLSs found in low-GC firmicutes revealed that these elements are mostly AT-rich, and frequently found close to the stop codon of genes located upstream. Typical rho-independent transcriptional terminators are relatively short SLSs, in which GC-rich stems made by 6 to 8 bp pairs are flanked on the 3' side by a stretch of 4 or more Ts [31-33]. To test the potential for SLSs from low-GC firmicutes to act as terminators, the distribution of As and Us at their *termini *was analyzed. Most (65 to 75%) of the higher stability SLSs located immediately downstream from annotated CDSs feature four Ts at their 3' border (Fig. 5, panel A, grey bars). The number of SLSs exhibiting the same features drops to 20%, or less, in other bacteria (Fig. 5, panels A and B). Interestingly, in low-GC firmicutes more than 50% of the SLSs featuring four Ts at the 3' border carry also four As at the 5' border (Fig. 5, panel A, black bars). Again, SLSs with identical features are 5- to 10- times less abundant in other bacteria (Fig. 5, panels A and B). The concomitant presence in low-GC firmicutes of 4As and 4Ts respectively at the 5' and 3' SLS *termini *is not merely due to the high AT content of their genome, but rather appears to be the result of some functional selection. In fact, very low numbers of SLSs with the inverted organization, namely carrying 5' 4Ts and 3' 4As, were found (see Fig. 5).

### Distribution of intergenic SLSs

**Figure 6:**
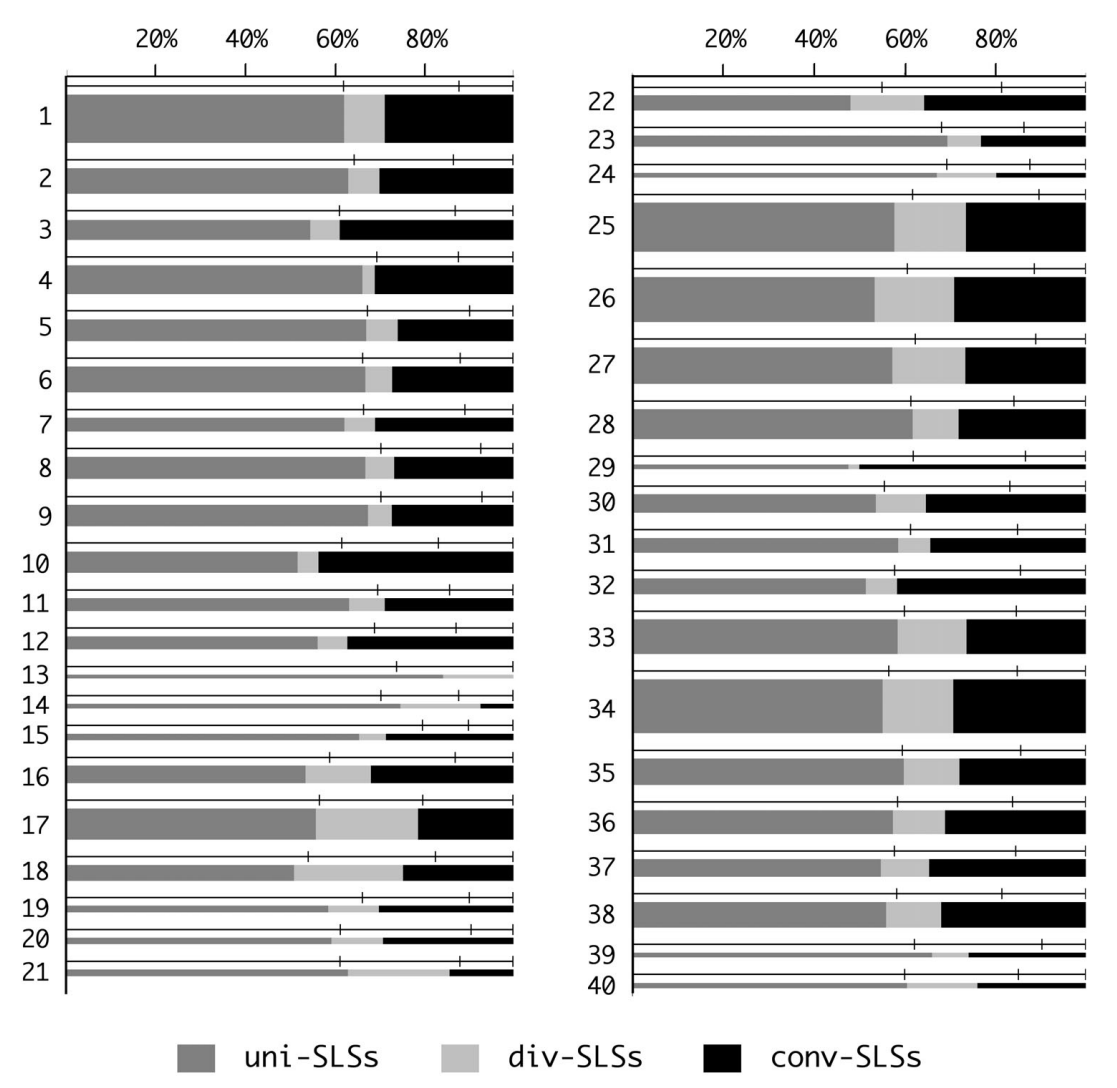
**Classes of intergenic SLSs**. Based on the orientation of flanking CDSs, higher stability intergenic SLSs (*dG *< -10 KCal/mole) have been sorted into three categories, as indicated at the bottom (see Results). The width of each stacked bar denotes the fraction of SLSs belonging to the three categories. The thickness of the bars is proportional to the cumulative sizes of IGRs (lengths below 25000 bp are not to scale, but are represented by a minimal bar width). Lines above bars represent the intergenic space, split by vertical dashes in three segments respectively corresponding, left to right, to the cumulative lengths of IGRs flanked by unidirectionally, divergently and convergently transcribed CDSs. According to the parameters adopted, no conv-IGS was found in the genome *of M. genitalium *(see row 13). Only IGRs ranging from 29 to 500 bp were taken into account, since smaller regions can not contain the shortest detectable SLSs, and bigger ones might derive from inaccurate genome annotation. Bacterial genomes are numbered 1 through to 40 as in Table 1.

**Figure 7:**
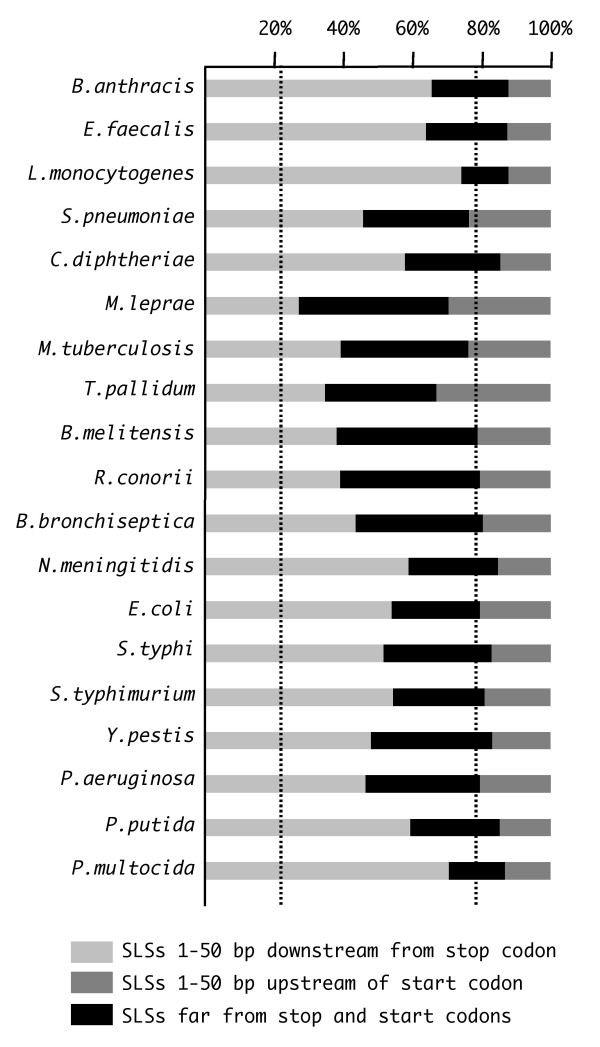
**Subsets of uni-SLSs**. SLSs located between unidirectionally transcribed CDSs have been subdivided into three categories, relatively to their distance from flanking CDSs as indicated at the bottom. The position of the dotted lines across bars denotes the averaged amount of intergenic space occupied by the three categories of SLSs analyzed. The uni-IGS minimum size was raised to 129 bp, since shorter IGSs cannot be assigned to any of the three categories. SLSs were selected as in Fig. 6.

The relative positions of higher stability SLSs within the IGRs were analyzed in all the species listed in Table 1. Based on the orientation of flanking CDSs, IGRs were combined (Fig. 6) to form three intergenic spaces (IGS): a) uni-IGS, between CDSs transcribed unidirectionally, i.e. along the same orientation; b) conv-IGS, between convergently transcribed CDSs; c) div-IGS, between divergently transcribed CDSs. SLSs falling within each intergenic space are accordingly referred to as uni-, conv- and div-SLSs. In all species uni-SLSs are the largest (around 60%) SLS fraction, but no enrichment is observed, as their number reflects the length of the uni-IGS. In contrast conv-SLSs, which represent 20 to 30% of total intergenic SLSs, are concentrated in a much smaller space, as the corresponding conv-IGS covers 8 to 12% of the overall intergenic space in practically all tested species. Conversely, div-IGS, which covers 25–35% of the intergenic space, only hosts about 10% of SLSs. A corollary of this distribution is that SLSs tend to favour, as a preferential location, the 3'- over the 5'- end of flanking CDSs. To test this hypothesis also on the uni-SLSs, a representative set of these regions were further sub-divided into three sub-regions corresponding to the two 50 base spans named *left *and *right*, respectively close to 3'- and 5'-ends of the flanking CDSs, and the remaining, variable length, intermediate subregion, named *center*. Short IGRs, which could not be split into appropriate subregions, were not included in the analysis. Similarly a small number of extremely long regions, which might derive from inaccurate genome annotation, were not used. The number of SLSs found in the described subregions (Fig. 7) shows that also the uni-SLSs clearly favour the 3'-end location: in the vast majority of species SLSs found within left subregions outnumber by 2- to 4-fold those found in the equally long right subregions.

### SLSs spanning repetitive DNA elements

**Figure 8:**
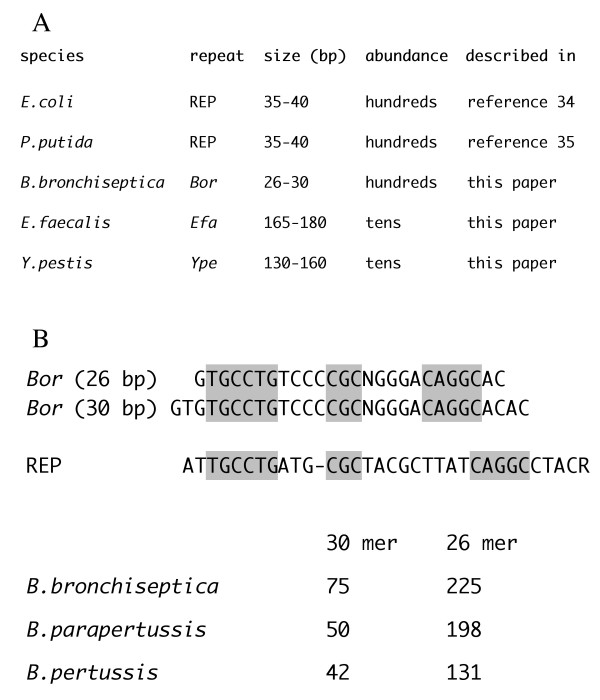
**SLSs and sequence repeats**. **A) **Repeated DNA families spanning SLSs identified in different prokaryotic species are listed. **B) **The consensus sequence of the *Bordetella *Bor repeats is shown at the top. Sequence identities with the *E.coli *REP z1 sub-type are highlighted. N, any nucleotide; R, purine. The relative abundance of the 26- and 30-bp long members of the *Bor *family in sequenced *Bordetella *genomes is reported.

Some intergenic SLSs may coincide with, or be a modular component of repeated DNA families. The clustering of intergenic SLS at the 3'-end of genes opens the possibility that this relative enrichment may be related to a functional role, not necessarily connected to termination. A search for DNA repeats, known from literature to cluster at the 3'-end of coding regions, revealed that REPs (repetitive extragenic palindromic sequences) found in *E. coli *[34] and *P. putida *[35] are a component of the selected population of 3'-end clustered SLS. By using SLSs as BLAST query sequences, we could identify repeated, previously undescribed DNA elements in various species (Fig. 8A). The *Bor *repeats are short SLSs ranging in size from 26 to 30 bp over-represented in *Bordetellae*. The 30-mer is found in numbers ranging from 42 to 75 in different *Bordetella *species, whereas the smaller 26 bp core is much more abundant (Fig. 8B). *Bor *are found close to coding regions, and share some similarity with the *E. coli *REPs (Fig. 8A). Novel DNA sequence elements, larger than REPs were found in *Y. pestis *and *E. faecalis *(*Ype *and *Efa *elements, respectively: Fig. 8A). Members of both DNA families tend also to be preferentially inserted close to the 3'-end of annotated CDSs.

## Discussion

The ability of a DNA or an RNA segment to fold into a stem-loop structure derives from the presence of complementary bases, and such segments stochastically occur in every large sequence, no matter the origin, even randomly generated, provided that some level of balanced distribution of nucleotides within single strand is guaranteed. This is certainly true in bacterial genome sequences, where oligonucleotide distribution reveals compositional symmetries in a variety of complete genomes [36,37]. The problem of evaluating the relevance of a particular motif in terms of the likelihood of generating it by chance in a given sequence has been extensively faced (see for example the work by Robin and coworkers [38] for the probability of finding a motif composed of two 'boxes' separated by a variable distance). Here we chose an 'experimental' approach, based on randomized genomes produced by reshuffling the natural one, with two types of constraints: preservation of a variety of k-let frequencies and a more complex model where genic and intergenic regions are separately shuffled with conservation of aminoacid sequence and codon usage. SLSs found in naturally occurring genomes clearly outnumber those expected from the result of similar analysis in their randomized counterparts (Figs. 2 and 3). It appears that natural genomes somehow tend to favour the formation of specific sets of stem-loop structures, typically the more stable ones. These sets significantly contribute to the higher SLS numbers observed in naturally occurring genomes, compared to their random counterparts. The phenomenon has been observed in bacterial genomes which widely differ in terms of size, GC content, evolutionary relatedness. Data are in agreement with literature reports, showing that, in large-scale analyses of prokaryotic mRNA populations, coding regions had a significant bias toward more local secondary structure potential than expected [15].

The evolutionary pressure promoting the potential formation of stem-loop structures at genome-wide level may serve different functional purposes. At the DNA level, stem-loop structures may play a role in replication, transcription, and recombination. However, as the vast majority of prokaryotic genomes is composed of expressed, protein-coding, regions, the contribution to mRNA secondary structure formation should be taken into account for most SLSs, especially those including G-U pairs. Most SLSs fall within coding regions (Fig. 1), in agreement with their size, which typically exceeds those of non-coding regions by a factor of ten. Still, when evaluating their significance, ribosome coverage and formation of secondary structures within protein-coding regions should be regarded as alternative, ribosomes being expected to prevent the formation of most low stability mRNA structures. Higher stability structures may however result in translational pausing, possibly used in regulatory mechanisms such as attenuation [16]. In specific instances, coding SLSs correspond either to remnants of transposon-like sequences [30], or to regions encoding repetitive protein domains, such as those found in the mycobacterial PE genes or in anchored cell-wall proteins conserved in several microorganisms (not shown).

Although less numerous, SLSs tend to be more frequent within the much smaller IGRs, where a typical bias towards energy levels and genome localization may be observed, highly indicative of specific, non-random, SLS subpopulations. All the analyzed low-GC firmicutes feature a marked enrichment in higher stability intergenic SLSs. Both structure and genomic location suggest that most of these sequences may function as rho-independent transcriptional terminators. The finding is not surprising *per se*, since the transcriptional factor *rho *is not essential in *Bacillus subtilis *and *Staphylococcus aureus*, and other Gram-positive bacteria with a low GC-content lack a *rho *homolog [39,40]. However, SLSs found in low-GC firmicutes are atypical as transcriptional terminators, as most of them carry, in addition to the canonical 3' U-rich tract, a complementary A-rich tract at the 5'-end (Fig. 5). This arrangement is known not to impair termination as, for example, in the *E. coli thr *operon attenuator, the terminator features a GC-rich stem-loop flanked by 9 Us at the 3'-, and 6 As at the 5'-end, and site-directed mutagenesis has shown that upstream adenines are neither essential, nor detrimental to transcription termination [41]. The 4A/4T containing SLSs found in low-GC firmicutes, when located at a short distance from convergently transcribed genes, may function as bi-directional terminators [42]. Alternatively, these AU-rich SLSs may serve additional functions, such as mRNA stabilization, as point mutations in transcription terminators are known to affect the stability of upstream RNA segments [43,44].

Bacteria other than low-GC firmicutes do not feature similar AT-rich terminator-like structures, still the distribution of SLSs within IGRs is clearly non random. When the frequency of SLSs is analyzed according to the type of IGR, all bacteria show a strong preference for SLSs within convergent, i.e. flanking the 3'-end of CDSs, rather than divergent IGRs (Fig. 6). Furthermore within unidirectional IGRs, higher stability intergenic SLSs are also preferentially found within the 50 bp tract immediately following the stop codon of the neighbouring CDS (Fig. 7). This distribution strongly favours the notion that most higher stability intergenic SLSs are transcribed, and may therefore function at the RNA level. Although termination is the expected role for a large fraction of them, especially in bacteria where *rho *dependent termination is not relevant, their number and the observed sequence features leave open the possibility of additional roles, such as RNA stabilization, translational regulation by riboswitches and attenuators [19,16]. Alternatively these SLS may be targeted by specific nucleases and rapidly degraded, thus functioning as RNA instability determinants. Finally, it must be recalled that some intergenic SLSs may be transcribed independently of the flanking genes. In recent years several groups provided support to the notion that prokaryotic intergenic sequences encode a variety of small, non-coding (nc) RNAs fulfilling diverse functions [reviewed [45]]. It will be of interest to assess whether selected intergenic SLSs may lead to the identification of novel nc-RNAs in RNA populations.

Some SLSs show strong similarity with each other, and may be grouped into families of repetitive sequences. Here we describe *Bor *sequences (Fig. 8), a set of palindromic elements, over-represented in all *Bordetellae*, which recall in length and sequence the *E. coli *REP sequences. *Bor *containing RNA may fold into hairpins similar to REP RNA, and possibly play an analogous role, i.e. the stabilization of the cotranscribed mRNA [34]. The larger *Ype *and *Efa *elements (Fig. 8) are members of less numerous DNA families spread in the genomes of *Y. pestis *and *E. faecalis*, respectively. These sequences are similar in size and abundance to other intergenic repeats, such as NEMIS in *N. meningitidis *[22] and ERIC in *Yersiniae *[23], which are cotranscribed with flanking genes and may fold into similarly organized RNA hairpins. Preliminary data indicate that both *Ype *and *Efa *RNA elements may indeed enhance the stability of cotranscribed mRNA sequences [De Gregorio E, Silvestro G and Di Nocera PP, unpublished results]. Quantitatively, members of these families only account for a small fraction of intergenic SLSs. As revealed by a preliminary BLAST analyses (not shown), further substantial similarities may be detected within the identified SLSs. Each of these families may therefore be extended, by including more elements sharing sequence similarity, but not initially found because of the presence of defective, or less pronounced, secondary structures. Further work will be necessary to eventually obtain a systematic classification of bacterial DNA families spanning, or coinciding with SLSs.

## Conclusion

An in-depth analysis of SLS features and distribution was carried out in 40 different bacterial species. Data suggest that an evolutionary pressure preserved specific non random populations of higher stability SLSs in most of the analyzed genomes. Many of these sequences are plausibly transcribed, and may be involved in transcriptional and/or post-transcriptional control. Specific SLS containing sequences are members of three previously undescribed families of repeated sequences found in *Yersiniae*, *Bordetellae *and *Enterococci*.

## Methods

### Genomic sequence data

Complete genomic sequences and their annotations about CDS, rRNA and tRNA were downloaded from the online repository made available at The Institute for Genomic Research (TIGR). Automatic annotations have been stored into a SQL database (SLS-DB), for further analysis. PostgreSQL has been used as the SQL Database Management System [46], according to techniques previously described [47,48].

### SLS identification

SLS identification was performed by using the program *rnamotif *of the package RNAMOTIF, version 2.1.2 [49] according to the following rules:

-GU pairing in the stem was allowed

-the minimal stem length was 12 bp

-loop length could vary from 5 to 100 nt

-1 bulged or 1 mispaired base, at least two matches away from the ends of the stem, was allowed.

As a consequence of the constraints imposed, the smallest SLS that could be found is 29 bp. Due to the allowance for GU pairing, *rnamotif *had to be run on both strands of the input sequence. Completely overlapping SLSs were discarded by 6 runs of the *rmprune *tool, also from the RNAMOTIF package.

The Gibbs free energy (*dG*) of each SLS containing region was calculated by calling the built-in function *efn2 *of *rnamotif*. The minimum free energy with no constraint for SLS formation was obtained by running the program *mfold *developed by Zuker and coworkers [50] on the SLS sequences.

### SLS pre-filtering

When two or more SLSs where found overlapping, only the most stable one was counted.

### Intergenic regions

Intergenic regions (IGRs) were derived, stored and annotated into the SLS-DB, according to the ORF collection provided by TIGR. For some tests, IGRs of size ranging from 29 to 500 bp were selected (see also legend to Figure 6).

### Shuffled genomes

The program *Shufflet *[51] was used to generate random sequences and to shuffle bacterial genomes by preserving k-lets of different lengths. In order to shuffle sequences with k-let higher than 6, *Shufflet *was compiled by setting the variable MAXORDER to 15. An alternative shuffling method (referred to as DS in Fig. 3), was used to take into account the information about protein coding sequences. Basically coding regions were shuffled by using the program *Dicodonshuffle *[15], while *Shufflet *set to k-let = 2 was used for non coding regions.

## Abbreviations

bp, base pair

nt, nucleotide

Mb, megabase

## Authors' contributions

MP created the pipeline for identification and automatic annotation of SLSs within bacterial sequences and contributed to sequence and statistical analysis. GS retrieved the sequences and other informations and provided manual annotation and analysis. AB contributed to the development of the pipeline. PPDN and GP conceived and coordinated the study. All authors read and approved the final manuscript.
